# Stabilizers of edaravone aqueous solution and their action mechanisms. 1. Sodium bisulfite

**DOI:** 10.3164/jcbn.17-61

**Published:** 2017-10-26

**Authors:** Masahiko Tanaka, Natsuhiko Sugimura, Akio Fujisawa, Yorihiro Yamamoto

**Affiliations:** 1 School of Bioscience and Biotechnology, Tokyo University of Technology, 1404-1 Katakura-cho, Hachioji, Tokyo 192-0982, Japan; 2 Materials Characterization Central Laboratory, Waseda University, 3-4-1 Ohkubo, Shinjyuku-ku, Tokyo 169-8555, Japan

**Keywords:** edaravone, sodium bisulfite, bisulfite adduct, edaravone trimer, hydrogen peroxide

## Abstract

Edaravone (3-methyl-1-phenyl-2-pyrazolin-5-one) has been used as a free radical scavenging drug for the treatment of acute ischemic stroke in Japan since 2001. Edaravone is given to patients intravenously; therefore, it is distributed in the form of an aqueous solution. However, aqueous solutions of edaravone are very unstable because it is present as edaravone anion, which is capable of transferring an electron to free radicals including oxygen, and becomes edaravone radical. We observed the formation of hydrogen peroxide and edaravone trimer when aqueous edaravone solution was kept at 60°C for 4 weeks. We proposed the mechanism of edaravone trimer formation from edaravone radicals. Lowering the pH and deoxygenation can effectively increase the stability of aqueous edaravone solution, since the former reduces edaravone anion concentration and the latter inhibits edaravone radical formation. Addition of sodium bisulfite partially stabilized aqueous edaravone solutions and partially inhibited the formation of edaravone trimer. Formation of bisulfite adduct was suggested by ^13^C NMR and HPLC studies. Therefore, the stabilizing effect of sodium bisulfite is ascribed to the formation of a bisulfite adduct of edaravone and, consequently, reduction in the concentration of edaravone anion.

## Introduction

**Fig. 1:**
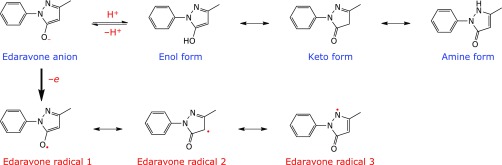
Chemical structure of edaravone and its anion, and the formation of edaravone radical.

Edaravone (3-methyl-1-phenyl-2-pyrazolin-5-one, Fig. 1) has been used as a free radical scavenging drug for the treatment of acute ischemic stroke in Japan since 2001.^(1)^ Recently, edaravone was approved for the treatment of patients with amyotrophic lateral sclerosis (ALS) in Japan (2015) and the USA (2017).^(1)^ We assume that its scavenging activity against peroxynitrite is of greater importance in ALS treatment.^(2)^

Edaravone is given to patients intravenously, therefore, it is distributed in the form of an ampoule or bag containing the aqueous edaravone solution.^(3)^ The solid keto form of edaravone is very stable, but because edaravone is present as the enol form and its anion in water, its aqueous solution is very unstable. Both enol and anion are present as equal concentrations at pH 7, since the pKa of edaravone is 7.0.^(4)^ Edaravone anion has been shown to be an active form with antioxidant activity, as it can donate an electron to free radicals including molecular oxygen (Fig. 1).^(4)^ This results in the formation of a relatively stable edaravone radical consisting of 3 resonance structures (Fig. 1).^(4)^

To increase the stability of aqueous edaravone solution, lowering the pH and deoxygenation are well-established methods, as the former reduces the concentration of edaravone anion and the latter prevents electron transfer from edaravone anion to oxygen. Moreover, sodium bisulfite and cysteine are used as stabilizers,^(3)^ but the precise mechanisms have not been elucidated to date. In this paper, we examined the effect of sodium bisulfite on the stability of aqueous edaravone solution. In order to investigate the effect of sodium bisulfite itself, we did not lower the pH of the solution or deoxygenate the sample. We confirmed that sodium bisulfite partially stabilized the aqueous edaravone solution and inhibited the formation of degradation products of edaravone to some extent. The formation of bisulfite adducts was suggested by ^13^C NMR and HPLC studies. Therefore, the stabilizing effect of sodium bisulfite is ascribed to the formation of edaravone-bisulfite adducts and, consequently, reduction in the concentration of edaravone anion.

## Materials and Methods

### Chemicals

Edaravone, sodium bisulfite, and other chemicals were of the highest grade commercially available. Water was purified with the Milli-Q Advantage system (Merck Millipore, Tokyo, Japan).

### Stability of edaravone in aqueous solution

**Fig. 2:**
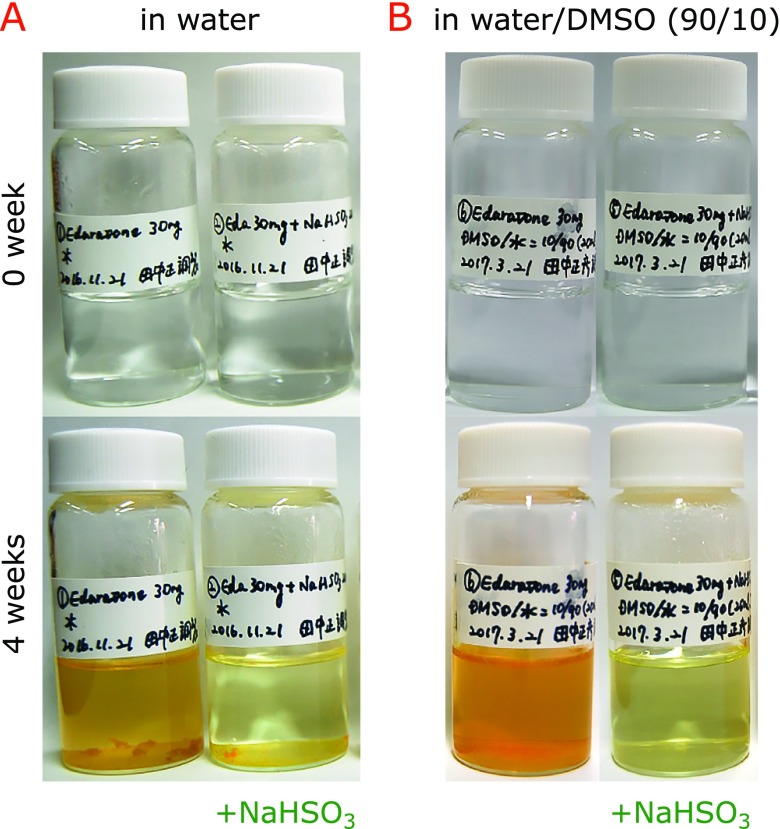
Precipitate formation during the storage of 8.61 mM edaravone in water (A) and in aqueous 10% DMSO (B) in the absence and the presence of 9.61 mM sodium bisulfite at 60°C for 4 weeks.

 Edaravone (30 mg) was dissolved in 20 ml of water. If necessary, 1 N aqueous NaOH was added, and then the final pH was adjusted to 5–6 by adding 1 N aqueous HCl. The resulting 8.61 mM edaravone aqueous solution was mixed with 0 or 20 mg NaHSO_3_ (9.61 mM). Also, edaravone (30 mg) was dissolved in 2 ml of dimethylsulfoxide (DMSO) and mixed with 18 ml of water containing 0 or 20 mg NaHSO_3_. The above 4 solutions were placed in screw capped 50 ml vials and kept at 60°C for 4 weeks. To eliminate the effect of room light, the vials were covered by aluminum foil. Every week, pictures of the vials were taken (Fig. 2) and edaravone concentrations were measured by HPLC without removing precipitate if formed.

### HPLC analysis

Edaravone was quantified by HPLC separation on an CAPCELL PAK ADME column (5 µm, 4.6 × 250 mm, Shiseido, Tokyo, Japan) using methanol/40 mM aqueous NaHPO_4_ (60/40 by volume) as the mobile phase (0.5 ml/min) with detection at 295 nm. Edaravone anion and sulfite adduct were measured by HPLC separation on an aminopropylsilyl column (LCNH_2_, 5 µm, 4.6 × 250 mm, Supelco Japan, Tokyo) using methanol/acetonitrile/40 mM aqueous NaHPO_4_ (45/50/5 by volume) as the mobile phase (1 ml/min) with detection at 240 nm.

### LC/time-of-flight mass spectrometry (TOFMS) analysis

The precipitate was dissolved in methanol and analyzed by a reverse phase HPLC system combined with TOFMS (JMS-T100LC, JEOL Ltd., Tokyo, Japan). The mobile phase was 60% methanol containing formic acid (2.6 mM) and was delivered at a rate of 1.0 ml/min. An octadecylsilyl column (Wako, Osaka, Japan; 5 µm, 4.6 mm × 250 mm) was used for separation. Elution of metabolites was monitored by absorption at 210 nm using a UV detector tandemly connected to TOFMS. Then, 1/4 of the eluent was induced into TOFMS. Negative ionization was performed at an ionization potential of −2,000 V. The optimized applied voltages to the ring lens, outer orifice, inner orifice, and ion guide were −5 V, −10 V, −5 V and −500 V, respectively. To obtain accurate *m/z* values, trifluoroacetic acid was used as an internal standard for *m/z* calibration.

### NMR analysis

^1^H and ^13^C NMR spectra were recorded on a Bruker Avance 600 spectrometer with BBO 600 MHz S3 5 mm probe (Bruker Biospin, Rheinstetten, Germany). All measurements were carried out at 25°C, and relaxation times were set at 1 s for ^1^H and 2 s for ^13^C.

### Detection of hydrogen peroxide

Hydrogen peroxide was measured using a specific HPLC method with isoluminol detection, as described previously.^(5)^ The above LCNH_2_ column was employed and methanol/40 mM aqueous NaHPO_4_ (90/10 by volume) was used as the mobile phase (1 ml/min).

## Results and Discussion

### Precipitation during storage

**Fig. 3:**
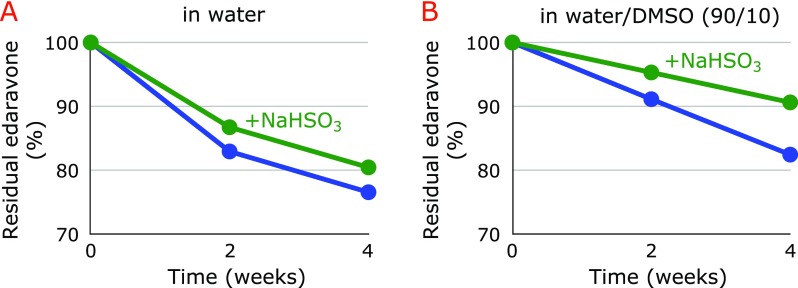
Changes in edaravone concentration during the storage of 8.61 mM edaravone in water (A) and in aqueous 10% DMSO (B) in the absence and the presence of 9.61 mM sodium bisulfite at 60°C for 4 weeks.

Figure 2A shows the formation of a precipitate during the storage of 8.61 mM edaravone in water at 60°C for 4 weeks. More than 20% of edaravone decomposed during storage (Fig. 3A). Addition of 9.61 mM sodium bisulfite partially prevented the precipitate formation (Fig. 2A) and the degradation of edaravone (Fig. 3A) as compared with sodium bisulfite-free conditions. Addition of 10% DMSO did not affect the above results (Figs. 2B and 3B).

### Formation of edaravone trimer

**Fig. 4:**
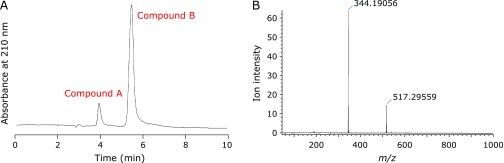
Reversed-phase HPLC chromatogram of the precipitate monitored at 210 nm (A) and the MS spectrum of compound B (B).

Next, we isolated the precipitate and subjected it to LC/TOFMS analysis. Figure 4A shows the reversed-phase HPLC chromatogram of the precipitate monitored at 210 nm, indicating the precipitate contained two compounds. The *m/z* value of compound A with the shorter retention time was determined to be −173.10, which is identical with that of edaravone −173.10). Moreover, authentic edaravone gave identical retention time with compound A. Therefore, we concluded that compound A is edaravone. The *m/z* value of compound B with the longer retention time was determined to be −517.30 (Fig. 4B) and was identified as edaravone trimer [4,4-bis-(3-methyl-5-oxo-1-phenyl-2-pyrazolin-4-yl)-3-methyl-1-phenyl-2-pyrazolin-5-one, monoisotopic mass-1 = −517.20]. The fragment of −344.19 was assigned as edaravone dimer [4,4'-bis(3-methyl-1-phenyl-2-pyrazolin-5-one) anion radical (monoisotopic mass-1 = −344.13)]. The missing fragment (−517.30 + 344.19 = −173.11) was assumed to be non-anionic edaravone radical. It is, therefore, reasonable that the above fragment was not detectable at the negative mode of detection. These results also support that compound B is edaravone trimer.

**Fig. 5:**
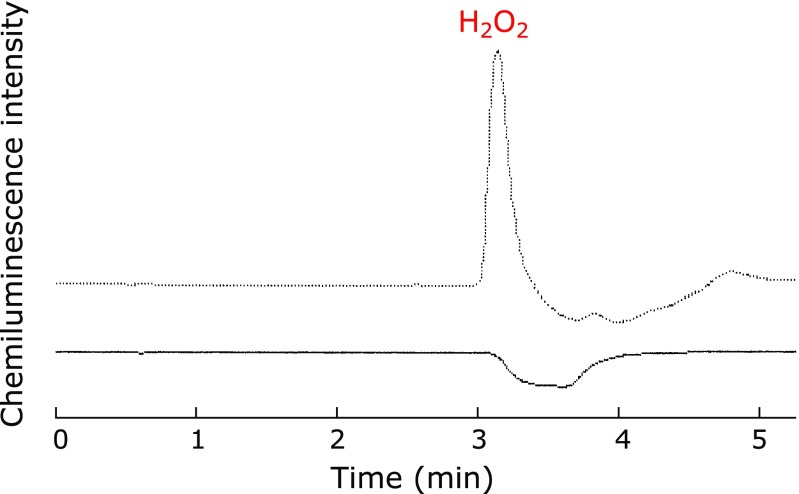
Detection of hydrogen peroxide before (solid line) and after (dotted line) the incubation of 8.61 mM edaravone in water at 60°C for 4 weeks.

The formation of edaravone trimer was observed in the reaction of edaravone with DPPH^(6)^ or phenoxyl radical^(7)^ in the absence of oxygen, indicating that edaravone radical is the precursor of edaravone trimer. In our experimental conditions, oxygen is consumed by edaravone anion, producing edaravone radical and superoxide. Superoxide disproportionates to hydrogen peroxide and oxygen. In fact, the formation of hydrogen peroxide (at least 6 µM) was confirmed by specific HPLC/chemiluminescence detection^(5)^ (Fig. 5). Elution of edaravone gave a negative peak since it quenched background chemiluminescence (Fig. 5).

### Formation mechanism of edaravone trimer

**Fig. 6:**
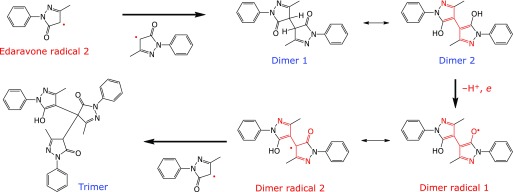
Formation of edaravone trimer from edaravone radical 2 through edaravone dimer.

We propose the following mechanism of edaravone trimer formation from edaravone radical 2 (Fig. 6). The combination of two molecules of edaravone radical 2 gives edaravone dimer 1, which is then converted to the much more stable conjugated edaravone dimer 2 by enolization. Dimer 2 is easily converted to dimer radical 1, since dimer radical 1 is very stable due to conjugation with 4 double bonds. Dimer radical 1 is rearranged to dimer radical 2 and it gives edaravone trimer by combination with edaravone radical 2. It is noteworthy that the yield of edaravone dimer was very small compared to that of edaravone trimer. In fact, there were no significant peaks between edaravone and edaravone trimer (Fig. 4A). This result can be ascribed to the fact that edaravone dimer is much more reactive than edaravone.

### ^13^C NMR spectrum of edaravone

**Fig. 7:**
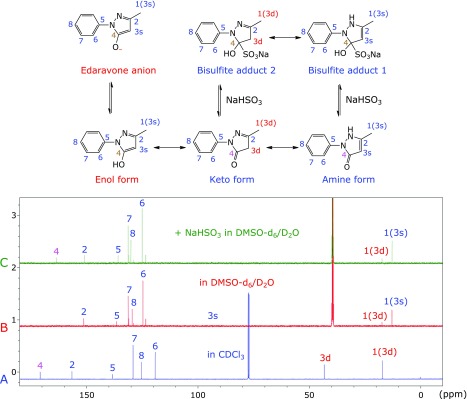
^13^C NMR spectra of edaravone in CDCl_3_ (A), in DMSO-d_6_/D_2_O = 10/90 (v/v) (B), and in the presence of NaHSO_3_ in DMSO-d_6_/D_2_O = 10/90 (v/v) (C).

The keto form of edaravone is believed to be the most stable form in non-protic solvents. In fact, ^13^C NMR spectrum of edaravone in CDCl_3_ shows eight single peaks, which are assigned as shown in Fig. 7A, and support the keto form of edaravone. When the solvent was changed to 10% DMSO-d_6_ in heavy water (D_2_O), the spectrum was drastically changed, as shown in Fig. 7B. The chemical shifts of carbons 3 and 4 disappeared, the chemical shift of carbons 1, 6, 7 and 8 were moved and split, and the chemical shift of carbon 2 was also changed. These results indicate that edaravone has at least two different forms in water, most likely the enol and edaravone anion forms in addition to the keto form. Interestingly, the chemical shift of carbon 1 was affected by the condition of carbon 3. Therefore, we named carbon 1 with methylene carbon 3 as 1(3d) and carbon 1 with methine carbon 3 as 1(3s). Since the peak height of carbon 1(3s) was higher than that of carbon 1(3d), the enol and edaravone anion forms were more predominant than the keto form. This result is acceptable because the solvent consisting of 10% DMSO-d_6_ in D_2_O is protic and protic solvents stabilize the enol form of edaravone, and thus the enol form liberates proton and edaravone anion. Moreover, Heteronuclear Multiple Bond Coherence (HMBC) measurement revealed carbon 3 existed at 92 ppm as methine (3s) rather than methylene (3d) at 42 ppm. This result is consistent with the above notion that the enol and edaravone anion forms were predominant in water.

### Addition of sodium bisulfite

Sodium bisulfite reacts with carbonyl to give the sulfite adduct,^(8,9)^ as shown in Fig. 7. Since the keto and the amine forms of edaravone have carbonyl groups, two bisulfite adducts are possible. The addition of sodium bisulfite did not change the chemical shifts of edaravone carbons, except that the chemical shift of carbon 4 reappeared, but at a different position, as shown in Fig. 7C. These results indicate that the bisulfite adducts liberate carbonyl to some extent and the carbonyl should be different from the carbonyl located in the keto form of edaravone. Therefore, the amine form and its bisulfite adduct 1 should be predominant, which is consistent with the observation that the peak height of carbon 1(3s) was greater than that of 1(3d). This may be reflected by the stabilization of bisulfite adduct 1 by 6-membered ring (H-N-N-C-S-O^−^) intramolecular hydrogen bonding.

### HPLC detection of edaravone anion and bisulfite adduct

**Fig. 8:**
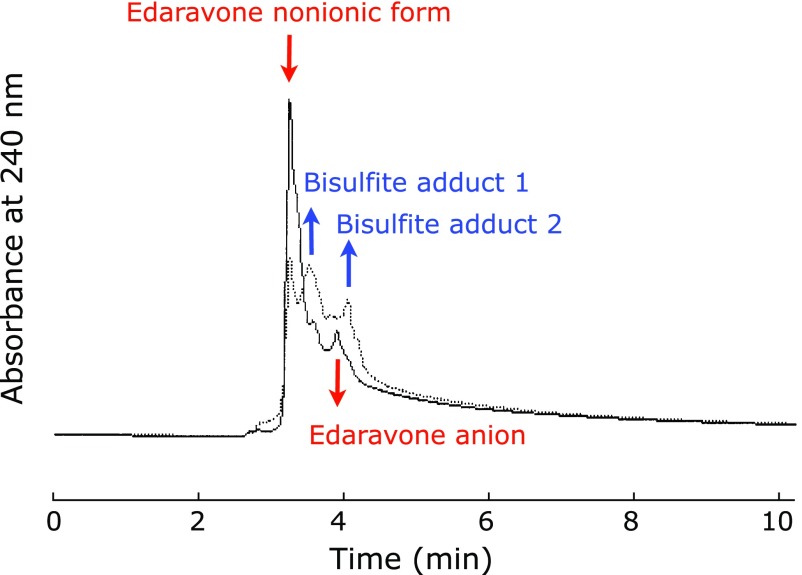
HPLC chromatograms of edaravone before (solid line) and after (dotted line) the addition of sodium bisulfite. An aminopropylsilyl column was employed and methanol/acetonitrile/40 mM aqueous NaHPO_4_ (45/50/5 by volume) was used as the mobile phase (1 ml/min) with detection at 240 nm.

Next, we injected edaravone solution with and without sodium bisulfite in order to have chromatographic evidence of their adduct formation. The solid line in Fig. 8 shows the chromatogram when an aqueous edaravone was injected, showing two peaks. Since the LCNH_2_ retains cation groups on the surface of the stationary phase, it has a stronger interaction with the edaravone anion than the non-ionic forms of edaravone. Therefore, the peak at 3.9 min should be edaravone anion and the peak at 3.2 min should be the non-ionic form of edaravone.

The dotted line in Fig. 8 shows the chromatogram after the addition of sodium bisulfite to an aqueous edaravone solution. The peaks at 3.2 and 3.9 min were decreased and the two peaks at 3.5 and 4.1 min were increased. These observations are consistent with the amount of non-ionic forms of edaravone being decreased by bisulfite addition and the two anionic sulfite adducts increased. The peak at 3.5 min was greater that that at 4.1 min. Therefore, the former and the latter are likely to be bisulfite adduct 1 and bisulfite adduct 2, respectively. This is consistent with the prediction that bisulfite adduct 1 is less polar than bisulfite adduct 2 due to the intramolecular hydrogen bonding.

**Fig. 9:**
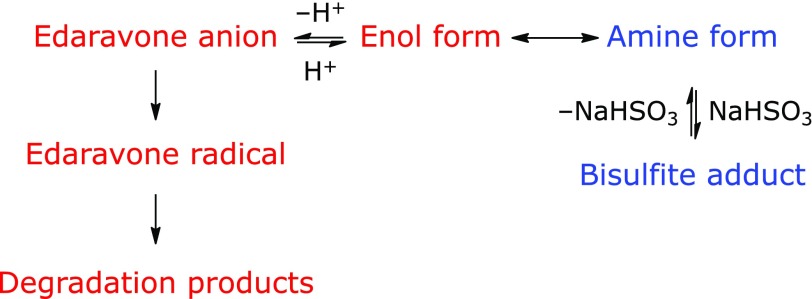
Effect of sodium bisulfite addition to aqueous edaravone.

Figure 9 summarizes that aqueous edaravone exist as edaravone anion, which tends to become edaravone radical after the donation of an electron. Edaravone radical is the precursor of degradation products. To increase edaravone stability, it is necessary to reduce the concentration of edaravone anion. The addition of sodium bisulfite is effective in reducing edaravone anion concentration, since the equilibrium shifts to the right and bisulfite adduct is produced. However, the efficacy of this method is limited because these reactions are under equilibrium and it is difficult to remove edaravone anion completely. We intend to describe how to stabilize the edaravone anion in a subsequent paper.

## Conclusion

Edaravone aqueous solution is unstable because of the presence of edaravone anion, which is capable of donating an electron to free radicals including oxygen and produces edaravone radical. We elucidated the mechanism of edaravone trimer formation from edaravone radicals. The addition of sodium bisulfite partially stabilized the aqueous edaravone solution and partially inhibited the formation of edaravone trimer. The formation of the bisulfite adduct was suggested by ^13^C NMR and HPLC studies, and this consequently reduces the concentration of edaravone anion.

## Abbreviations

ALS: amyotrophic lateral sclerosis
DMSO: dimethylsulfoxide
LCNH_2_: aminopropylsilyl column
TOFMS: time-of-flight mass spectrometry
